# The hallmarks of living systems: towards creating artificial cells

**DOI:** 10.1098/rsfs.2018.0023

**Published:** 2018-08-17

**Authors:** N. Amy Yewdall, Alexander F. Mason, Jan C. M. van Hest

**Affiliations:** Eindhoven University of Technology, PO Box 513 (STO 3.31), Eindhoven, MB, The Netherlands

**Keywords:** artificial cell, compartmentalization, energy transduction, information processing, division, adaptability

## Abstract

Despite the astonishing diversity and complexity of living systems, they all share five common hallmarks: compartmentalization, growth and division, information processing, energy transduction and adaptability. In this review, we give not only examples of how cells satisfy these requirements for life and the ways in which it is possible to emulate these characteristics in engineered platforms, but also the gaps that remain to be bridged. The bottom-up synthesis of life-like systems continues to be driven forward by the advent of new technologies, by the discovery of biological phenomena through their transplantation to experimentally simpler constructs and by providing insights into one of the oldest questions posed by mankind, the origin of life on Earth.

## 1. Introduction

**Figure 1.**
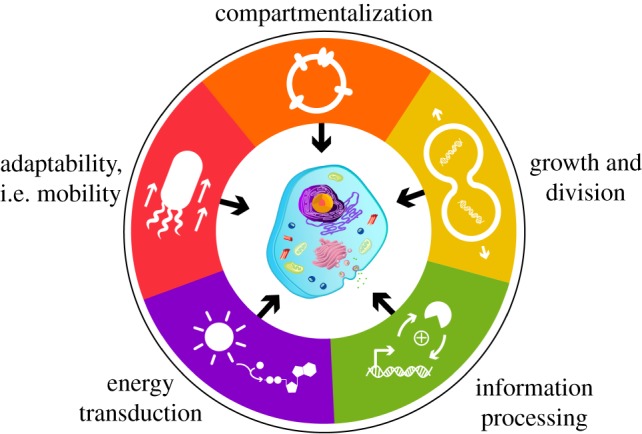
The hallmarks of life. A summary of the five characteristics required for systems to live and thrive. In the last decade, developments in bio-analogous and bio-mimetic bottom-up technologies have emulated aspects of each hallmark to inform us about the functional mechanisms behind each process, and about the opportunities to construct integrated artificial cell platforms.

With billions of years of evolution shaping the diverse living systems present today, we have only begun to comprehend the fascinating complexity and nuances of what makes living systems tick. Despite the huge variation among all forms of life, there are certain common characteristics that enable life to exist, and even thrive. We have defined these five characteristics as: compartmentalization, growth and division, information processing, energy transduction and adaptability (figure 1).

Advances in cellular biology have enriched our understanding of these characteristics and act as inspiration for the creation of systems that mimic life. Feynman's words of ‘what I cannot create, I do not understand’ lie at the heart of artificial cell research, especially concerning bottom-up technologies. Creating complex, multi-component systems uses convergent approaches among the different fields of biophysics, polymer engineering, protein modification, DNA nanotechnology and dynamic covalent chemistry. Here, we describe various ways in which bottom-up technology has addressed the hallmarks of living systems, and also identify the divide between what we can create and what nature can create, providing inspiration for future research.

## 2. Compartmentalization

The requirement of life to be compartmentalized is widely accepted as fact. Compartmentalization results in the concentration of reactants and chemical networks, protection of biological components from harsh external environments and the confinement of genetic material. Plasma membranes are semi-permeable and highly functional, allowing cells to interact with their environment, adopt specific shapes, display hierarchical order (organelles) and create temporary gradients to function under out-of-equilibrium conditions. Researchers have become increasingly adept at mimicking these properties using a wide range of materials, including phospholipids, block copolymers, colloids and protein–polymer hybrids. The many examples of these different types of bio-mimetic compartments have been summarized recently in several excellent reviews, and will not be described herein [1–5]. Instead, this section will focus on the functional aspects of the cell membrane that remain challenging to imitate.

### 2.1. Membrane-active elements at compartment interfaces

The cell membrane, as traditionally described by the fluid mosaic model, is a two-dimensional fluid composed of phospholipids in which membrane proteins are randomly dispersed [6,7]. In reality, membrane proteins make up a much larger mass fraction of the interface and are organized in tightly controlled ways. For example, 25% of the cross-sectional area in the plasma membrane is proteinaceous [8], and the ratio of protein to lipid is 76% in the inner mitochondrial membrane. Reconstitution of purified membrane proteins into synthetic compartments [9,10] results in artificial cells with membranes more akin to the fluid mosaic model than nature, with relatively low protein incorporation yields, and a dearth of complexity, using only one or two proteins. In the following discussion, several solutions to this limitation will be outlined.

**Figure 2.**
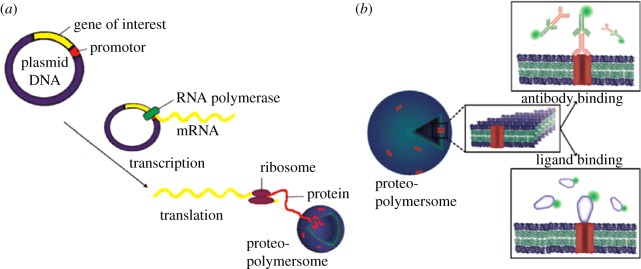
Schematic of G-protein-coupled receptor proteopolymersomes formed using IVTT. (*a*) Spontaneous insertion of G-protein-coupled receptors into polymersomes during cell-free expression. (*b*) Antibody and ligand-binding studies indicate that the G-protein-coupled receptor is reconstituted in an active conformation. Reproduced with permission from Wiley [15].

One possibility is to use cell-free protein synthesis for the incorporation of membrane proteins. *In vitro* transcription translation (IVTT) kits have revolutionized the field of synthetic biology by providing a reliable, customizable tool for the expression of proteins in entirely synthetic contexts. The cell-free expression of membrane proteins in the presence of liposomes directly facilitates their incorporation into the membrane in an active conformation [11–13]. The wide applicability of this technique was demonstrated with the expression of 85 different endogenous *Escherichia coli* membrane proteins using IVTT in liposomes [14]. Polymer membranes are also compatible with IVTT (figure 2), with the expression of Claudin-2 [16], G-protein-coupled receptors [15,17] and light-harvesting complex II [18] in polymersomes. However, as both liposomes and polymersomes are static compartments, they lack waste removal or recycling machinery, setting an upper limit to the amount of protein that can be produced before the concentration of inhibitory by-products reaches toxic levels [19]. A direct consequence of this is a limit on the number of different proteins that can be expressed in a single IVTT reaction, as the maximum concentration of active protein decreases for each additional template DNA added to the reaction. This particular issue can be circumvented by performing the IVTT reaction in a microfluidic device that enables by-product removal and reagent supplementation [20], albeit currently still restricted to the expression of soluble proteins due to the lack of a model membrane.

In order for an artificial cell to be able to effectively mimic the higher order behaviours observed in nature, such as energy transduction and communication, it must incorporate membrane-active elements to couple processes inside the cell to the outside world. As such, the challenges lie not only in increasing the amount and variety of functional membrane-bound entities, but also in developing a better understanding of how they interact with each other, cytosolic elements and the membrane itself.

### 2.2. Membrane complexity

Underlying the structural complexity of the plasma membrane is the arrangement of phospholipids within, exhibiting both lateral (liquid–liquid phase separation, or lipid rafts) [21] and transverse (different inner and outer leaflet) organization [22]. While, at present, engineering the energy-dependent systems responsible for maintaining this complexity is beyond our capabilities, significant advances have been made to mimic this complexity. Inducing lateral phase separation between liquid-ordered and liquid-disordered phases in model membranes has been important in the biophysical elucidation of lipid rafts [23]; however, the mechanisms driving such behaviour *in vivo* remain poorly understood. So, while it is possible to create lateral phase separation, it is not appropriate to call this organization, as the structures are thermodynamically defined. Transmembrane asymmetry has also been mimicked by inducing heterogeneity in synthetic compartments [24]. The time scale upon which heterogeneity is achieved for liposome-based systems using POPC is 5–6 h [25], as the small size of phospholipids enables their rapid redistribution. In contrast, polymer-based systems are metastable [26–28], and do not appear to change on measured time scales.

The organization of these elements in living cells is dynamic and adaptive, changing to suit a specific function in response to internal and external cues. Artificial cells at present are in thermodynamic equilibrium, and simply cannot mimic this behaviour, as these membrane characteristics are dictated by many regulatory pathways in a dissipative, far-from-equilibrium manner. The generation of (meta)stable complexity in membranes is nevertheless an important milestone, as it provides a set of tools to study biological processes in a controlled way.

### 2.3. Non-spherical morphologies

**Figure 3.**
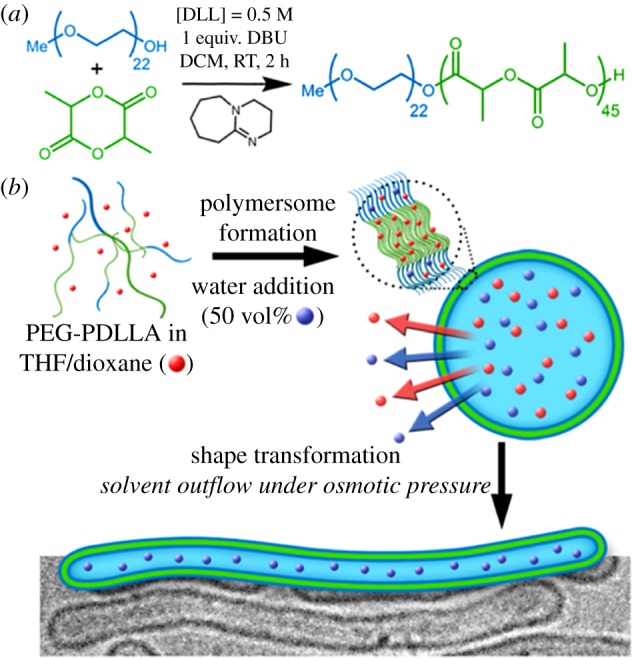
The osmotically induced formation of nanotubes from spherical polymersomes. DLL, D,L-lactide; DBU, 1,8-diazabicyclo[5.4.0]undec-7-ene; DCM, dichloromethane; RT, room temperature; PEG-PDDLA, poly(ethylene glycol)-poly(D,L-actide). Reproduced with permission from the American Chemical Society [30].

Many cells adopt non-spherical morphologies to adapt to their environment, or to perform certain functions. Almost every artificial cell model is a sphere, due to the minimization of interfacial energy. Shape transformations can be achieved using polymersomes via a number of different strategies, such as directional hydrophobic interactions [29] and osmotic shock [30,31] (figure 3). These shape changes are possible because polymer membranes are thermodynamically more stable, and ‘frozen’ in place once the transformative force is removed. It is more difficult to achieve non-spherical morphologies in liposomal systems for this very reason. Cells achieve shape differentiation by using an actin-based cytoskeletal network; this was reconstituted inside liposomes to study the effect of actin networks on membrane dynamics [32]. In a bioinspired approach, an artificial cytoskeleton constructed from DNA origami at the inner surface of liposomes drastically increased the mechanical stability of the system [33]. While both these examples show the effect of a subinterface network, the resulting morphologies were still spheres, thus more control is needed over the shape of the cytoskeletal mimic for shape control using this strategy. Combining lipids and polymers is another avenue only just starting to be explored, and it was recently demonstrated that the combination of lipids and polymers can result in the spontaneous formation of hybrid microtubules with phase-separated leaflets [34].

Cells also enter into highly controlled shape transformations during mitosis, where cell division is mediated by a network of proteins. Advances towards replicating this behaviour in artificial cells will be expanded upon in a later section.

### 2.4. Mimicking the cytosol

In recent years, it has become increasingly apparent that membrane-free organelles have an important role to play in many cellular processes [35,36], especially in bacteria where an open-plan structural architecture governs many internal processes [37]. Such examples found within nature include: chromatin organization with a cell, centrosome formation during division and long-term storage of proteins and genetic material in Balbiani bodies [38]. Their commonality is liquid–liquid phase separation resulting in a condensed phase and a dilute phase. Phase-separated liquid systems have minimum free energy, which enables the maintenance of a concentrated compartment without the constant input of energy by the cell [38]. Complex coacervates are synthetically simple and well-defined systems that can be used to mimic biological condensed phases, as they provide a dense, charged and crowded microenvironment amenable to the investigation and reconstitution of biological processes. For example, purified FtsZ protein polymerization can be induced in these liquid–liquid phase-separated microenvironments [39]. In addition, the IVTT processes were shown to be compatible in two different complex coacervate systems [40,41]. In one case, the crowded environment improved the rate of transcription [41], highlighting the capabilities of coacervates to mimic and investigate the effect of crowding on macromolecular processes.

**Figure 4.**
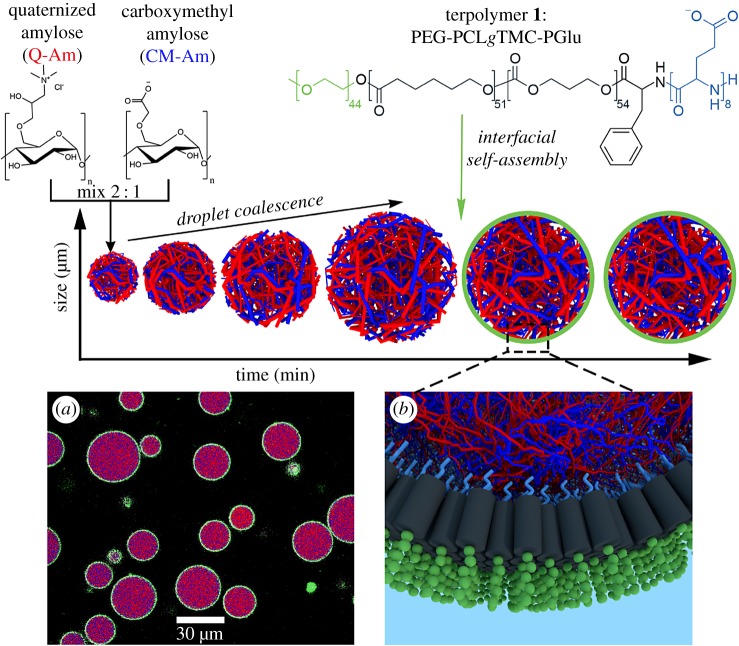
Hierarchical self-assembly of a terpolymer-stabilized coacervate protocell. Oppositely charged amylose biopolymers undergo complex coacervation and droplet formation, followed by interfacial self-assembly of terpolymer 1. (*a*) Confocal micrograph of terpolymer/coacervate protocells with internalized bovine serum albumin–fluorescein isothiocyante conjugate (purple) and terpolymer membrane (green, Nile Red). (*b*) Three-dimensional representation of the interfacial assembly of terpolymers. Reproduced with permission from the American Chemical Society [44].

However, the membrane-free nature of complex coacervates inhibits their use in experiments that take place on extended time scales. The main driving force for condensation, electrostatic interactions, also causes complex coacervate droplets to rapidly coalesce and lose structural relevance. To overcome this, coacervates can be stabilized using phospholipids [42], phosphotungstate polyanionic clusters [43] and a triblock copolymer [44]. This polymer forms a semi-permeable interface which enables the encapsulation of functional macromolecular cargo and indefinitely stabilizes the protocells to coalescence and content mixing (figure 4). Artificial cells based on these stabilized coacervate droplets are an exciting new platform because they offer several bio-mimetic properties in one system, namely a crowded inner phase, a membrane delineating the system from the outside world and a size of the same order as single cells.

One clear difference between complex coacervates and condensed phases found in nature is the lack of regulated assembly. Cells have the ability to exquisitely control the formation and dissipation of these phases in response to internal and external cues such as fluctuations in protein concentrations [38], yet the formation of complex coacervates currently lacks this regulatory complexity.

## 3. Growth and division

The generation of new cells is ultimately derived from growing and replicating existing cells. For this process to occur, compartments must be able to: (i) grow, (ii) deform and finally divide, as well as (iii) regulate all of this in time and space. Within living systems, the lipid bilayer and associated proteins that enable the coordination and control of these processes are pervasive for not only cell division, but also vesicular transport, organelle division and cellular movement [45]. Components involved in cellular growth and division can be reconstituted *in vitro*, which augments our understanding of their biological functions, and also inspires the synthesis of artificial cells.

### 3.1. Membrane synthesis and growth

The autonomous and heritable synthesis of membranes is not yet achievable for polymersome systems [46], so, for now, we are restricted to processes found in living systems. The easiest approach to growing membranes is the spontaneous insertion of de novo synthesized components into pre-formed vesicle membranes. Fatty acid vesicles have been heralded as possible pre-biotic compartments where growth and division are driven by the external environment [47–49]. These vesicles are dynamic and can grow by the spontaneous incorporation of new fatty acid moieties into pre-existing vesicles [50–54] or even by using a dipeptide catalyst to promote the recruitment of amphiphiles [55]. Primitive homeostasis was also established using fatty acid vesicles [56], where inhibitory moieties were diluted with subsequent vesicle divisions to restore RNA enzyme activity.

However, for the purposes of creating self-sustaining artificial cells, compartments should be based on more stable membranes, such as those composed of phospholipids as found in most living systems. In addition to increased stability, these membranes can form in a range of pH and salt conditions compatible with most proteins; phospholipid membranes also have unique permeability properties and, most importantly, the ability to be synthesized autonomously [3]. Autonomous membrane synthesis from amphiphilic precursors marks a crucial step towards self-sustained life-like growth. The generation of phospholipids within cells occurs via complex pathways where components and regulation vary between different cell types and organisms [57,58]. Simplified pathways from purified proteins have been reconstituted in liposome systems [59,60]. Alternative non-enzymatic approaches have exploited basic lipid chemistry and chemical catalysts to generate phospholipid membranes from amphiphilic precursors [53,54,61–63]. However, in both approaches, the final vesicle lipid composition lacks the diversity of different phospholipids normally present on cell membranes. This has been recently addressed by incorporating purified proteins to synthesize phospholipids that were efficiently incorporated into the membrane, growing both the size and complexity of the vesicles [64].

For a system to support life-like growth, the process of generating more membrane precursors must be not only internally driven, but also heritable. For this, membrane synthesis and growth must be coupled to the synthesizing machinery, and these proteins must be able to regenerate from genes. Simplified systems using cell-free protein expression have been reconstituted in both liposome [65,66] and coacervate systems [40], with limited success. The low efficiency of incorporation of newly synthesized phospholipids into the membranes resulted in a meagre increase in vesicle surface area. This disconnect between an efficient membrane growth and for these pathways to be heritable is the most obvious gap to bridge. However, this will require developments in approaches to mimic the complex cellular information-processing networks, as discussed later.

Mimicking membrane complexity with proteins such as flippases, as outlined above, as well as recreating the precise spatio-temporal regulation required to coordinate cell growth are both ongoing aspirations. Cell growth must also take into account accompanying structural features often associated with the lipid bilayer, such as cell walls or extracellular matrices, as these structures often impart shape and altered compartment permeability [45].

### 3.2. Altering membrane morphology and division

For life-like behaviour, artificial cells should possess an autonomous and heritable means to alter their membrane morphology, leading to division. Bottom-up technologies have, thus far, exploited many passive processes to generate daughter compartments in a thermodynamically driven manner. Membrane morphologies can be manipulated by altering the external environment of lipid or polymersome compartments, such as by changing salt concentrations [67], increasing membrane growth [63,68] and using osmotic shock [69,70]. Internalized RNA and altering Mg^2+^ ion concentrations can destabilize fatty acid membrane morphology and induce ‘division’ [52,63]. There are numerous examples where amphipathic helices insert into membranes, causing curvature that results in fission [71–74]. In fact, protein crowding on the membrane surface was recently shown to be sufficient for spontaneous division [75]. Regulation and spatial coordination of these passive processes will be a difficult endeavour, and, for artificial cells, internally driven processes would be more appealing.

**Figure 5.**
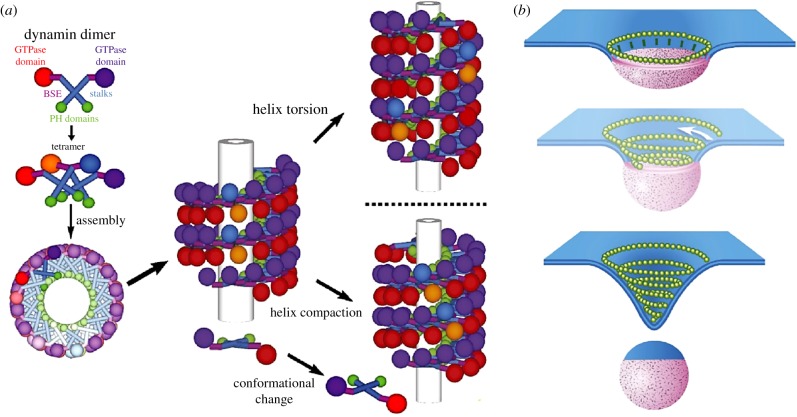
The mechanoenzymes involved in membrane scission. (*a*) The helical arrangement of the dynamin dimers along the surface of a protein tube changes with protein conformation change to further constrict the membrane. (*b*) The proposed model for membrane constriction mediated by spiralling ESCRT proteins (in yellow). BSE, bundle signalling element; PH, pleckstrin homology. Reproduced with permission from the National Academy of Sciences [76] and eLife Science Publications [77].

Within living systems, processes for membrane deformation and scission are usually actively directed by proteins, and *in vitro* reconstitution of these systems in liposomes has not only provided insights into their mechanism of action but has also paved the way to creating out-of-equilibrium cellular organizations within artificial cells. These active mechanisms are powered by protein complexes that consume either adenosine triphosphate (ATP) or guanosine triphosphate (GTP) to generate the forces required for membrane deformation and scission [45]. Constriction of the membrane by mechanoenzyme complexes is one mechanism by which active membrane scission occurs (figure 5), such as with the dynamin/BAR domain proteins [76,78] and the endosomal sorting complexes required for transport (ESCRT) [79,80]. Dynamins are GTPases that bind to the external surface of lipid membranes to facilitate the formation of vesicles, and their polymerization may be assisted by BAR domain proteins [45]. Dynamin proteins polymerize as helical structures causing the membrane to deform into tubes, and, upon GTP hydrolysis, the protein conformation changes to further constrict the membrane, which leads to fission [78] (figure 5*a*). By contrast, fission of membranes orchestrated by ESCRT protein complexes occurs when these proteins form dynamic spiral structures on the internal membrane surface, the mechanism of which is still under debate [45] (figure 5*b*). ESCRT complexes facilitate the fission of membranes that were previously connected by the cytoplasm, such as during cell division, and its localization is mediated via specific protein adaptors [79]. An alternative method of scission was proposed to be driven by friction, where BAR protein complexes form organized scaffolds around the outer surface of membranes to facilitate microtubule-dependent motor proteins pulling off vesicles [74,81].

Thus far, membrane fission systems largely consist of purified proteins reconstituted *in vitro*. These components will eventually have to be internally produced using encapsulated IVTT systems in order for these systems to be heritable. The molecular tool box to sculpt membranes has been expanded to include DNA origami, which can be considered an orthogonal approach to heritable membrane manipulating systems [82,83].

The shape of the membrane surrounding cells is often governed by additional structures beyond their phospholipid bilayer. For instance, plant and bacterial cells have an outer cell wall composed of polysaccharides, which help control the final shape of the cells, and prevent them from osmotic shock. FtsZ proteins were shown to not only constrict the lipid membrane [84,85] but also coordinate peptidoglycan synthesis in bacteria during cytokinesis [86,87]. The shape of a cell often reflects its particular function [37], and controlling morphology is a possible means for us to dictate the function of any future artificial cell.

### 3.3. Spatial organization for correct division

Evidence of spatial organization can be found within all levels of biological complexity. Active processes, where energy or matter is exchanged, drive these organized states far from thermodynamic equilibrium. Spatial organization of cellular components is critical for proper cell function and division. To mimic cellular spatial organization, a simplified strategy is to target proteins to membrane surfaces using either histidine-tagged proteins to nickel-nitrilotriacetic acid (NTA) modified surfaces [88] or by covalent targeting of molecules [89]. Unlike processes in nature, these bottom-up technologies cannot self-regulate, but are the first steps towards creating more complex spatial organizations.

Concentration gradients within a cell and polarization ensure not only equal splitting of resources and genetic material into two daughter cells, but also the correct positioning of the protein machinery required for division [37]. Diffusion is unreliable at equally partitioning molecules of low abundance into daughter cells. Nature's solutions involve materials actively segregated into opposite poles of rod-shaped bacteria by scaffold proteins [90] or by evenly distributing plasmid DNA using oscillating proteins ParA/B [91]. This phenomenon has yet to be replicated in synthetic compartments.

Establishing the middle of a cell is of crucial importance for correct division, and the bacterial Min protein system is a striking example where protein oscillations specify a statistical location within the cell. Min proteins oscillate via a reaction–diffusion mechanism on the inner surface of cell membranes, resulting in an average enrichment of MinD at the cell poles. MinD inhibits *E. coli* FtsZ assembly at the poles of the cell; however, the low MinD distribution at the midcell enables FtsZ to form the Z ring, thereby constricting the membrane and peptidoglycan synthesis initiates at the correct location [87]. Patterns of oscillating Min proteins have been observed *in vitro* using supported lipid bilayers [92–94], as well as in confined geometries [95]. Coupling these oscillations in synthetic compartments with the protein machinery required for membrane deformations described above is still an unmet goal in creating artificial cells capable of division [96].

## 4. Information processing

Information processing encompasses cellular responses to both internal and external signals. Intracellular information processing concerns the primary information carrier molecules made from nucleic acids, such as DNA and RNA, which are heritable. The replication and processing of nucleic acids is often performed by their output, which can either be proteins or segments of nucleic acids [97], resulting in a feedback system where output can influence the input of the system. These internal information-processing systems must also be responsive to extracellular cues. At the interface of compartments, cells are able to not only sense their surroundings and communicate, but also adapt their behaviour in response to these signals. The signals involved in this extracellular communication can extend beyond nucleic acids to include small molecules, proteins and peptides, and entire vesicles. Engineering a system where a signal triggers feedback that leads to a change in systems behaviour is vital for developing evolvable artificial cells that respond to both environmental and internal changes. The possibilities to mimic cellular information processing are broad and engineered examples exist at all levels of complexity from nucleic acids and proteins, to within protein metabolic pathways, to signal transduction and processing that occurs at the membrane.

### 4.1. Intracellular information processing: reconstituting transcription and translation in an artificial context

In addition to DNA, heritable information carriers in bottom-up technologies can be based on RNA [98–100] and other functional polymers, such as peptide nucleic acid [101]. These efforts reflect possible pre-biotic scenarios where alternative nucleic acid-like polymers, other than DNA, could have evolved to be the main information carrier of living systems. Through directed evolution, proteins have been developed to recognize and process these polymers [102], making these synthetic polymers and their protein processors a possible complementary information-processing system for future artificial cells.

For now, synthetic information-processing systems are dominated by DNA-based genetic networks that expand the engineering repertoire of living cells from all branches of life [103], but, when coupled with IVTT, can provide a means of regulated information processing inside artificial cells [104]. Away from the crowded and complex environs of cellular interiors, complicated genetic circuits and logic gates can be designed to feed back and regulate information processing.

**Figure 6.**
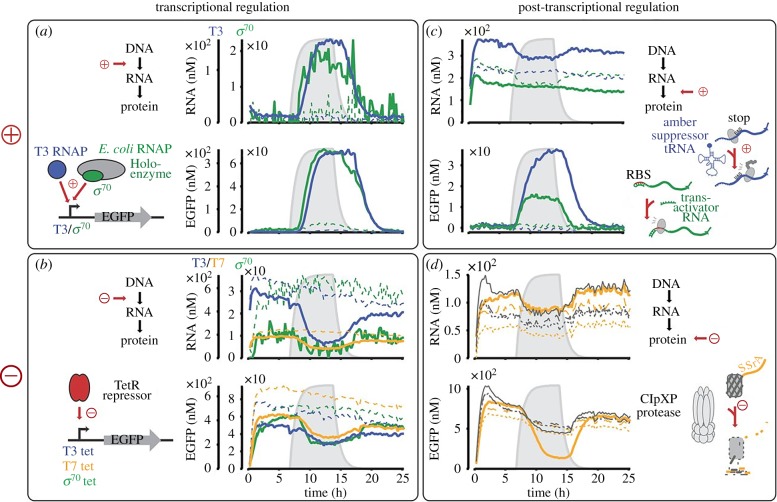
Cell-free transcription and translation systems enable the expansion of engineered genetic circuits, to produce regulatory feedback networks for artificial cells. Regulation can be at the transcriptional or post-transcriptional level using proteins or RNA. Reproduced with permission from the National Academy of Sciences [110].

Simple protocells can be created by capturing IVTT inside synthetic compartments, of which there are numerous examples using liposomes [105–109] and coacervates [40,41]. These systems mostly involve the expression of single gene products, where output may not necessarily influence input. Genetic circuits with regulatory feedback networks have been created (figure 6), and some in the form of logic gates [111]. These systems are capable of displaying more complex behaviours such as bi-stable switches [112], tailorable deactivation [113] and oscillations [110,114–118].

An appealing feature of encapsulated IVTT systems is their allure of eventual self-regeneration, in order for these systems to be hereditary. Although there are many components that constitute IVTT systems, the *in vitro* transcription of ribosomes is a promising first step towards regeneration [119]. A drawback of compartment encapsulation is their stochastic and low efficiency of incorporating all of the cell-free transcription/translation systems, which leads to variable protein production [120]. This must be addressed either using active targeting of components into the compartments or using sorting/amplification of successfully incorporated compartments to enrich the population. Nonetheless, our ability to link phenotype to genotype is a promising means to incorporate evolution elements into future artificial cells [121].

### 4.2. Extracellular communication: signal processing through the membrane

Beyond the internal processes of individual cells, information processing also encompasses internal responses to external signals that result in altered behaviours. Semi-permeable membranes permit the diffusion of external cues into compartments, and several protocellular membranes are capable of this [5]. Specialized membrane pores can be embedded into the compartment surface to provide additional regulation [122], such as the selective permeability of nutrients [108]; to generate proton gradients [123]; or even to generate peptide-based pores that conduct ions and bind blockers [124]. Adaptive regulation can be incorporated in these protein-based pores through covalent modification, for example by the attachment of a peptide to OmpF protein to open and close the pore as a function of pH [125] or redox environment [126]. For selective information processing, however, cells use membrane receptors that trigger internal signalling cascades. Replicating these complex cascades in bottom-up protocells is yet to be achieved, but preliminary steps have been taken to embed membrane receptors into artificial surfaces [16].

**Figure 7.**
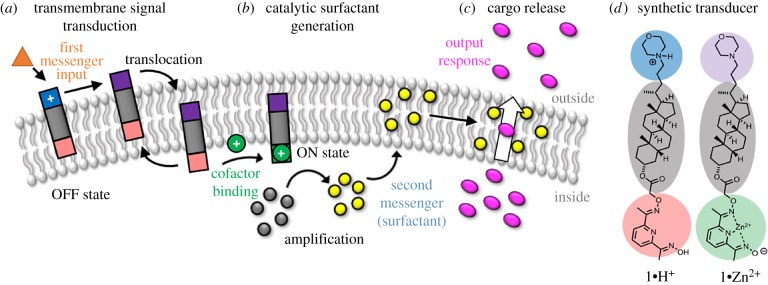
Bio-analogous membrane receptors that cause triggered release of cargo in response to an external signal. This process is mediated by hydrophobic small molecules rather than membrane proteins and provides an excellent alternative route to engineering artificial cell mimics. Reprinted with permission from the American Chemical Society [131].

Membrane proteins are notoriously difficult to work with [127], which makes bio-analogous synthetic molecules appealing alternatives that mimic information processing at compartment interfaces. For example, proton-pumping proteins can be mimicked with small molecules, using light-activated donor–acceptor triads and electron shuttles [128]. The behaviour of ion channels can be replicated with synthetic ionophores, hydrophobic molecules that selectively bind and release specific ions [129]. Signal transduction across a membrane resulting in a controlled release in response to a signal has also been achieved [130,131] (figure 7).

Extracellular communication of protocells with living cells has also been demonstrated [132–134]. Protocells that synthesized sugar were shown to communicate with live *Vibrio* cells [132], and these augmented quorum-sensing scenarios have also been replicated with *E. coli* [135]. Artificial cells were also shown to translate and communicate with *E. coli* cells [133]. So far, artificial cells augment the function of living systems, and this can be extended further by using synthetic compartments to capture living cells to form hybrid artificial cell systems [134,136].

Communication between two populations of artificial cells has been demonstrated for semi-permeable membrane systems, where signals diffuse and switch on internal cascades in a different protocell population [44,117,137]. Thus far, the information-processing systems in artificial cell communication are very simple pathways, with little regulation. Mimicking other forms of life-like communication, such as cell-to-cell vesicles or cell bridges, can be a way to achieve the complexity in regulation comparable to that found in nature, but often these processes require developments in membrane deformation technologies. Again, heritable information carrier systems are key for evolvable artificial cells.

## 5. Energy transduction and metabolism

In order to fuel processes necessary for life, cells must be capable of harnessing external sources of energy, such as solar energy from the Sun, and to be able to convert this into fuel sources to drive the biochemical reactions of metabolism. The main cellular energy currencies are ATP and NADH, and also FADH_2_ and GTP. These energy-rich small molecules are distributed throughout the cell to power metabolic pathways or to create new biomolecules. The ability to be self-sustaining distinguishes living systems from viruses or prions. From an artificial cell perspective, the incorporation of systems that enable the transduction of chemical or light energy into a source of cellular energy in a dissipative manner is an extremely important milestone on the path to a self-sufficient, life-like system.

### 5.1. ATP generation

ATP homeostasis within a cell is critical for the maintenance of out-of-equilibrium states. ATP is synthesized *in vivo* by a transmembrane protein called F_o_F_1_ ATP synthase (ATPase). ATPase is a rotary molecular machine that couples an electromotive force across the membrane into a rotational force, which in turn catalyses the formation of ATP from adenosine diphosphate and phosphate. For synthetic ATP generation, the ATPase must be coupled to a system that generates a transmembrane proton gradient, such as proton pumps localized to membranes. ATPase has been reconstituted in liposomes along with the light-activated proton gate bacteriorhodopsin [128] for light energy to drive ATP synthesis. Other components of the oxidative phosphorylation pathway have also been reconstituted with ATPase, such as cytochrome *c* oxidase [138] or cytochrome *bo*_3_ [139]. The chemical approach is to use a synthetic proton-transporting system which, upon photoexcitation, causes photoreduction of a proton shuttle and subsequent proton translocation across the membrane, for example, using a carotenoid–porphyrin–fullerene triad coupled with quinone [140].

**Figure 8.**
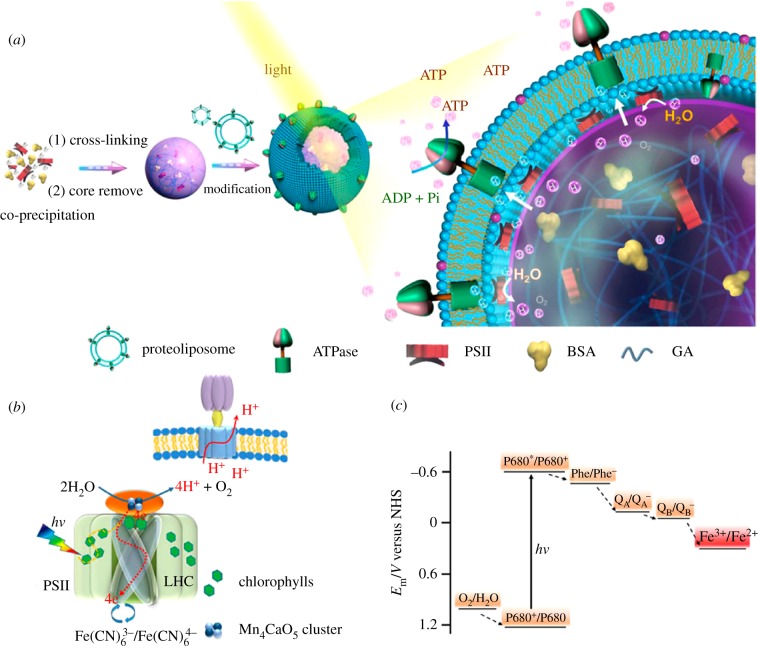
(*a*) Co-assembly of ATPase and PSII in lipid-coated microspheres results in the formation of artificial chloroplasts. (*b*) The proton gradient generated by PSII drives the rotation of ATPase. (*c*) Redox potential scheme of active elements in the system. ADP, adeosine diphosphate; Pi, inorganic phosphate. Reproduced with permission from the American Chemical Society [142].

Apart from liposomes, ATP generation has also been demonstrated in more structurally robust systems, such as in hydrogel-like microspheres [141,142] and polymersomes [143]. The creation of artificial chloroplasts capable of converting light energy into ATP was achieved using cross-linked photosystem II (PSII) protein microspheres covered in a lipid membrane containing ATPase (figure 8) [142]. In addition, encapsulated ATP-producing polymersomes in a protein-based foam were able to fix carbon upon illumination to imitate photosynthesis [144].

There are also a number of polymersome-based examples representing the generation of electromotive force across the membrane, using bacteriorhodopsin [145,146] and cytochrome *c* oxidase [147]. However, these were not coupled to ATPase. While ATP is the most attractive energy-rich molecule that can be produced in an artificial cell due to its ubiquity in nature, other small molecules involved in biomolecular energy transduction such as NAD(P)H and H_2_O_2_ can also be synthesized [148]; however, these are beyond the scope of the present article.

### 5.2. Metabolic pathways

The metabolism of cells comprises interconnected biochemical pathways that are responsible for the synthesis or breakdown of molecules, as well as energy homeostasis. Processes such as glycolysis, the citric acid cycle and oxidative phosphorylation all result in the production of energy-rich molecules, such as ATP and NADH, which can be used to fuel enzymes to catalyse downstream reactions. These pathways are often complex, involving many enzymes; therefore, it is worthwhile considering simplified pathways for engineering artificial cells. Simplified metabolic networks have been successfully reconstituted in synthetic compartments [108,149,150]. These reactions often consist of the downstream pathways fuelled by energy-rich molecules. That is, they lack the energy regeneration requirement for living systems.

Within the examples of diverse living systems, evolution has satisfied the need for metabolism using orthogonal enzymes or entirely different pathways [151]. These examples of convergent evolution can act to inspire the design of artificial cells. One such example is the completely synthetic carbon fixation pathway where 17 enzymes, from nine different organisms, were reconstituted *in vitro*, leading to improved fixation rates compared with *in vivo* pathways [152].

### 5.3. Dissipative systems

Life forms are dynamic, far-from-equilibrium systems that exhibit a constant flux of energy [153]. This is of course accomplished by the integration of the above two capabilities, energy transduction and metabolic pathways (energy utilization), within the same compartment. The concept of dissipative self-assembly is rapidly gaining in importance in the field of supramolecular chemistry [154–157], and will likely have a significant impact on many fields including artificial cell research. While the dynamic, dissipative cellular behaviours discussed in this review are currently beyond the scope of our capabilities, there are already a number of studies demonstrating rudimentary dissipative properties that could find use in bio-mimetic applications. For example, polymer vesicles with transient membrane permeability that is dependent on the addition of a fuel [158] could be used as adaptive organelle mimics. Transient fibres and gels fuelled by dimethylsulfate [159] or using a redox-active gelator [160] also bear a resemblance to actin fibres and microtubules. There is huge scope for research in this area, and the advent of more life-like artificial cell systems will surely be coupled with advances in our understanding of dissipative molecular self-assembly.

## 6. Motility as an example of adaptability

Adaptability is key for generating artificial cell systems. Motility can be considered a basic form of adaptability, as cells exhibit the ability to respond to their environment and move towards sources of energy, whether that is light (phototaxis) or chemical fuel (chemotaxis). This characteristic is useful for the survival of both unicellular and multi-cellular life, and as such is an interesting behaviour to mimic in bottom-up artificial cells because it involves the integration of two main systems: energy transduction and signal processing. In addition, motility is a property that is easy to observe on the macro-scale, and is a phenomenon that has been mimicked in both purely biological and chemical contexts. For an entity to achieve self-propulsion, it must convert a source of energy into kinetic energy. In addition, it should not do this in a stochastic manner; movement should ideally be directed towards a higher concentration of fuel in order to minimize the expenditure of energy and increase survival. This process thus involves signal sensing (determination of fuel position) and instruction (activation of motile elements to move there).

**Figure 9.**
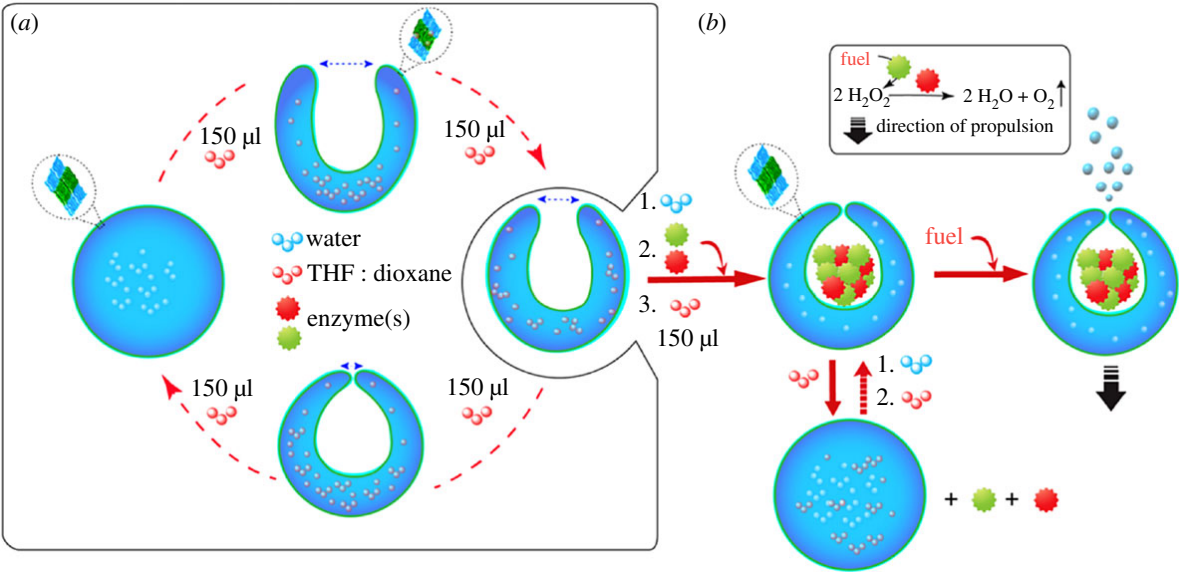
Schematic depiction of stomatocyte-based enzyme-driven nanomotors. (*a*) Solvent addition method for stomatocyte formation. (*b*) Assembly of the nanomotor with multiple enzymes entrapped inside the structure. The enzymes are responsible for generating the propelling jet of oxygen gas during the catalytic reaction. THF, tetrahydrofuran. Reproduced with permission from the American Chemical Society [149].

Movement generation in nature at the microorganism level can occur by several different modes, such as cilia or the bacterial flagellar rotor [161]. The overarching principle is the requirement for asymmetry, or non-reciprocal deformations, as the viscous forces of water dominate at this scale and inertia has a negligible effect [162]. Movement in artificial systems can, of course, be generated through external force fields, for example with magnetite nanoparticles in a magnetic field. However, with external forces at play, an entire population of particles will move as an ensemble. To achieve autonomous behaviour of individual entities, taxis must be generated at the molecule/particle level. Many strategies exist to achieve this [163,164], ranging from synthetic molecular motors [165] to bubble evolution on asymmetric inorganic nanoparticles [166]. An example of bubble-based propulsion are the stomatocyte-based nanomotors, where bowl-shaped structures made from polymers can encapsulate cargo, such as platinum nanoparticles [167] or catalase [149], to induce motility as oxygen gas is produced and forced out of a small aperture to generate a propulsive force (figure 9). These nanomotors could be used to mobilize future artificial cells.

However, these colloidal systems are stochastic in their behaviour, and even when there is an element of chemotaxis, for example with the directed motion of catalase towards hydrogen peroxide [168,169], the process is immediate with the catalyst acting directly in a gradient of ‘fuel’. This is in contrast with nature, where the sensing systems and the movement generation systems are separated in space and time. In order to truly mimic adaptability, these two components need to be integrated in the same system, using strategies described earlier in this article to equip artificial cells with the ability to transduce information (functional membranes), compute information (DNA/protein networks) and then generate motion (natural or artificial motors) in a specific direction. Motion generation could be achieved by the coupling of the aforementioned colloidal/molecular motors with artificial cell scaffolds. For example, an inorganic Ni/Ti helical flagellum attached to the surface of liposomes results in magnetically controllable motion [170]. Another approach would be the reconstitution of large, multi-component protein-based motors such as the flagellar motor. However, this complex is one of the largest found in nature, a 45 nm structure assembled from approximately 13 different subunits [161], and as such has not yet been wholly reconstituted in a model compartment. Certain components have been shown to retain activity, such as PomA/PomB, which are involved in torque generation [171]. These processes mimic basic unicellular motility, whereas in multi-cellular systems, via internal cytoskeletal proteins, generation of polarity and extracellular matrices play a key role in membrane deformation. The amalgamation of an energy transduction system and protein components required for membrane deformation and motility has been demonstrated [172], where ATP synthases were reconstituted in giant unilamellar vesicles (GUVs) and used to power the fibrillation of cytoskeletal proteins, actin and microtubules. Although shape deformation was not shown for these particular GUVs, it is known that, with the right liposome dimension and flexibility, actin filaments can interact with and deform membranes [173]. Light-activated ATP synthesis was recently demonstrated to induce actin-driven shape deformation in liposomes [174]. These examples are promising first steps towards coordinating different hallmarks to achieve more life-like adaptability.

Motility is only one example of adaptability. Living systems demonstrate an array of adaptable behaviours, such examples range from within single cells, where internal recycling mechanisms can be activated in response to stress [175], to populations of cells that exhibit collective adaptive behaviour [176,177]. These are all tantalizing end goals for artificial cell research.

## 7. Critical discussion

The creation of an artificial cell that satisfies all the hallmarks of life has not yet been achieved. We have made great progress in mimicking individual characteristics of life, and even combining several hallmarks into bottom-up technologies. However, many examples are in fact a rediscovery of colloidal effects in life-like contexts, and this acts as an important reminder that life indeed is based on core principles of physical chemistry. Our current efforts are thermodynamically limited, and truly life-like behaviours are attainable only with the incorporation of some form of metabolism and energy dissipation. Other key challenges in the pursuit of artificial life include controlling the spatio-temporal distribution of each nanoscale component, as well as addressing the compatibility of current artificial cell platforms to support all of the hallmarks of life. Addressing these is not trivial, and to have the best chance of success in achieving this complex goal, multiple and convergent bio-mimetic, bioinspired and bio-analogous approaches are required to one day create artificial unicellular organisms. As we become more advanced at mimicking the five hallmarks, integrating structures into living systems and moving towards evolvable life, ethical considerations must also be addressed, as the enthusiasm of scientists for this topic can also be met with societal criticism and resistance.

Although the authors acknowledge that simplified synthetic tissue systems have been created [177] and provide a useful platform for investigating cell-to-cell communication, the challenges associated with the bottom-up synthesis of multi-cellular systems, and even higher-order assemblies such as tissues and entire organisms, have not been addressed. Truly mimicking these systems by increasing hierarchical complexity requires exquisite control over communication, extracellular structures and other physiological characteristics beyond the scope of current technology.

Creating artificial living systems requires multi-disciplinary collaboration because a convergence of diverse approaches is needed to progress. Understanding the biophysics of living biological processes is critical for engineering systems from the bottom up. In addition, top-down synthetic biology can provide insights into the essential processes for living systems that will make artificial cells work. Computer science will enable us to engineer in the face of complexity as disciplines including, but not limited to, machine learning and neural networks map the plethora of interactions required for living processes. The five hallmarks of life are a means to simplify the complex, multi-faceted problem of creating artificial life into more tenable goals. When each of these hallmarks is addressed in unison such a system can satisfy NASA's working definition of life: ‘a self-sustaining chemical system capable of Darwinian evolution’.

## Data accessibility

This article has no additional data.
